# Engineering control circuits for molecular robots using synthetic biology

**DOI:** 10.1063/5.0020429

**Published:** 2020-10-01

**Authors:** Ting-Yen Wei, Warren C. Ruder

**Affiliations:** 1 Department of Bioengineering, University of Pittsburgh, Pittsburgh, Pennsylvania 15213, USA; 2 Department of Mechanical Engineering, Carnegie Mellon University, Pittsburgh, Pennsylvania 15213, USA

## Abstract

The integration of molecular robots and synthetic biology allows for the creation of sophisticated behaviors at the molecular level. Similar to the synergy between bioelectronics and soft robotics, synthetic biology provides control circuitry for molecular robots. By encoding perception-action modules within synthetic circuits, molecular machines can advance beyond repeating tasks to the incorporation of complex behaviors. In particular, cell-free synthetic biology provides biomolecular circuitry independent of living cells. This research update reviews the current progress in using synthetic biology as perception-action control modules in robots from molecular robots to macroscale robots. Additionally, it highlights recent developments in molecular robotics and cell-free synthetic biology and suggests their combined use as a necessity for future molecular robot development.

## INTRODUCTION

**FIG. 1.**
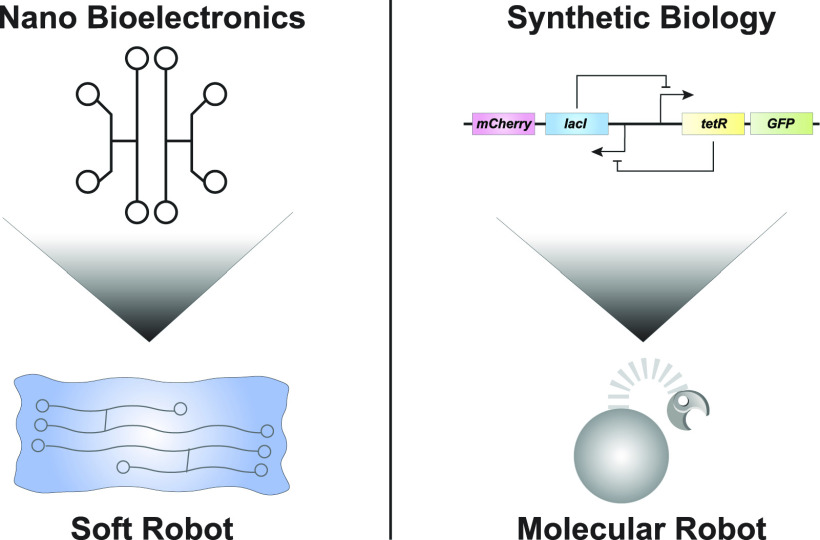
Control systems for soft robots and molecular robots. Similar to the synergy between bioelectronics and soft robots, synthetic biology could provide a control system for enabling perception-action behaviors in molecular robots.

Bioelectronics translates biological signals into electrical outputs, creating bio-electronic interfaces and enabling the successful development of biomedical devices such as pacemakers, deep-brain stimulators, and electronic skins.^1^ In order to increase sensitivity and biocompatibility, bioelectronic devices can be miniaturized to the nanometer scale of their biological counterparts.^1^ Miniaturized bioelectronic devices serve as sensors and act as core components in soft robotics, similar to the synergy between synthetic biology and molecular robotics [Fig. 1].

With advances in nanotechnology, a variety of nanomaterials, such as nanotubes^2,3^ and nanowires,^4^ have been developed and deployed to create wearable or implantable physiological monitoring and stimulation devices. The reduction in physical size improves comfort and increases device density. The small size of nanoscale sensors enables them to be deployed into objects with minimal invasiveness.^5^ In addition to their size, stretchability, biocompatibility, and self-healing capabilities are crucial factors when it comes to adhering these devices to human skin or tissue. Due to the trade-off between conductivity and stretchability, it is hard to fulfill these criteria simultaneously while maintaining robust electrical performance. Circuit design strategies have been introduced to improve the robustness and accuracy of bioelectronics.^2^

**FIG. 2.**
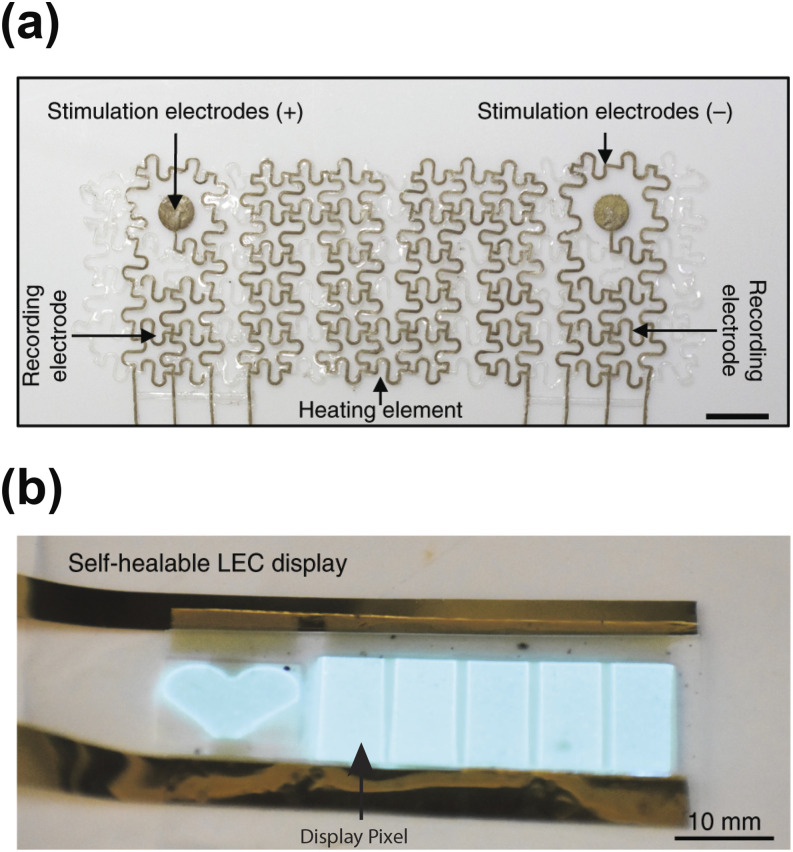
Bioelectronics as sensors in soft materials. (a) A stretchable and biocompatible ECG sensor consisting of Ag–Au core–sheath nanowires. The scale bar is 1 cm. Reproduced with permission from Choi *et al.*, Nat. Nanotechnol. **13**(11), 1048–1056 (2018). Copyright 2018 Springer Nature. (b) A self-healing, wearable electronic skin consisting of carbon nanotubes and polymers. The device can monitor ECG signals and provide visual feedback using the light emitting capacitor (LEC) display. Each display pixel represents a specific heart rate range. Reproduced with permission from Son *et al.*, Nat. Nanotechnol. **13**(11), 1057–1065 (2018). Copyright 2018 Springer Nature.

Alternatively, pairing nanoelectronics with soft materials can improve the stretchability and comfort of bioelectronic sensors.^6^ Choi and co-workers developed a stretchable and biocompatible nanowire composite consisting of gold-coated silver nanowires in elastomer matrices.^4^ While silver nanowires ensured conductivity and a gold-coated layer conferred biocompatibility, a poly(styrene-butadiene-styrene) elastomer layer was included to form a cushion-like microstructure that resulted in a soft and highly stretchable material. They utilized this material to build wearable and implantable bioelectronic devices to monitor electrophysiological signals^4^ [Fig. 2(a)].

Furthermore, incorporating soft materials grants self-healing abilities to bioelectronic sensors.^3,6^ Son and co-workers incorporated nanowires into a self-healing polymer matrix to create an electronic skin system.^3^ When damaged, the polymer matrix autonomously healed and reconstructed its conductive and mechanical properties. Using this self-healing material, Bao and co-workers built a multi-functional electronic skin system with an electrocardiogram sensor and a feedback display array. Figure 2(b) shows the display array with each display pixel representing a specific heart rate range. Thus, bioelectronics can provide sensor modules for soft robots, enabling the interaction between physiological signals, such as heartbeat and body temperature, and electrical systems. The integration could be especially useful for medical applications that require close physical contact with patients.^7^

While soft robots are used for monitoring electrical signals in human bodies, molecular robots can monitor biochemical, molecular signals inside human bodies. Robotics at nanoscale and microscale is a promising technology for applications such as active therapeutic delivery and minimally invasive surgery.^8,9^ The small size of molecular robots enables them to access previously unreachable areas throughout the human body, offering localized diagnosis and treatment with greater precision and efficiency.^8^ A molecular robot consists of three essential components: an actuator, a sensor, and a processor.^10,11^ Actuators power molecular robot actions. Sensors detect the environmental information, while processors analyze the gathered information and respond according to predetermined algorithms. The ability to sense, analyze, and react to complex environments, through a process in robotics known as a perception-action loop,^10^ is essential for building autonomous robotic systems. However, the functions of the current molecular machines are typically confined to repeating predetermined tasks without the ability to change their actions in response to novel external stimuli.

**FIG. 3.**
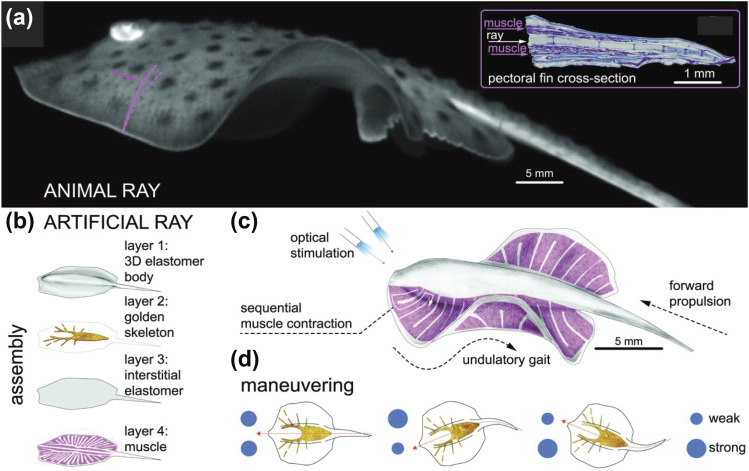
Genetically engineered cells as perception-action modules on a soft robot ray. (a) An image of an animal ray swimming and its musculoskeletal structure. (b) A soft robot ray consisting of four layers—two elastomer layers: one gold skeleton layer and one cardiomyocyte layer. (c) Cardiomyocytes were genetically engineered to contract in response to light. (d) By optically actuating right and left fins, the robot ray can swim and maneuver around obstacles. Reproduced with permission from Park *et al.*, Science **353**(6295), 158–162 (2016). Copyright 2016 The American Association for the Advancement of Science.

The ultimate goal of building autonomous robots is to combine sensing, computation, and action.^10^ Nature provides abundant examples of such autonomous systems, from macrophages chasing pathogens to animals preying on their next meal. Engineering onboard perception-action modules can influence and alter output behaviors. For example, Heyde and Ruder showed using the engineered microbiome in microbiome–host interactions to affect host behaviors.^12^ In a bio-inspired swimming robot ray developed by Park and co-workers, cardiomyocytes powered the actuation and served as a perception-action module.^13^ The way batoid fish swim is highly energy-efficient, which is a desirable trait in robotic systems. To mimic how batoid fish swim, they simulated their musculoskeletal structure by sandwiching a gold skeleton between two elastomer layers [Fig. 3(a)]. On the interstitial elastomer layer, fibronectin was printed to guide rat cardiomyocytes growing in a specific pattern similar to living ray muscles. With a single layer of heart muscle cells capable of downward contraction, the robot ray used the gold skeleton to actuate its fins, enabling chordwise front-to-rear undulatory motions [Fig. 3(b)]. To mimic the neural system controlling the sequential activation of fin muscles in real rays, the researchers genetically engineered cardiomyocytes to create heart muscle cells that only contracted in response to blue light [Fig. 3(c)]. Using genetically engineered cardiomyocytes, the robot ray swam at various speeds and maneuvered around obstacles by modulating light frequency and independently actuating right and left fins [Fig. 3(d)]. In the example of the ray, the cardiomyocytes provided the perception-action module, sensing light inputs (perception) and responding by waving the ray fins (action).

This research update will explore how synthetic biology can be integrated into nanomachines to build molecular robots. We will first briefly review the current progress of molecular machines and robots. Next, we will highlight the cell-free synthetic biology toolset and showcase how cell-free synthetic biology, in particular, can play an important role in future molecular robot developments. Furthermore, we will focus on the current progress in using synthetic biology tools as perception-action modules in robots at the macroscale and microscale.

## MOLECULAR MACHINES AND ROBOTS

Molecular machinery can be categorized into two major categories based on their composition: non-biological and biological molecular robots. Catenanes and rotaxanes, two primary molecular machines, have dominated the field of non-biological machinery since the 1990s.^14^ Catenanes consist of two interlocking rings with one ring gliding around the other ring. Rotaxanes consist of a cyclic molecule threaded onto an axle molecule. The structure of catenanes and rotaxanes enables motions of one component relative to the other component. The molecular dynamic properties of catenanes and rotaxanes have been exploited to build nanomotors,^15^ pumps,^16^ dissipative catalysts,^21^ and a chemical synthesizer.^22^

Imitating macroscopic mechanical machines at the molecular level can spark the development of molecular motors.^17^ A molecular motor functions by converting a particular type of energy into another. Biological molecular motors successfully generate energy using chemical gradients or the hydrolysis of adenosine triphosphate (ATP). In comparison, artificial molecular motors are now able to operate autonomously using chemical energy with a molecular motor developed by Wilson and co-workers.^15^

**FIG. 4.**
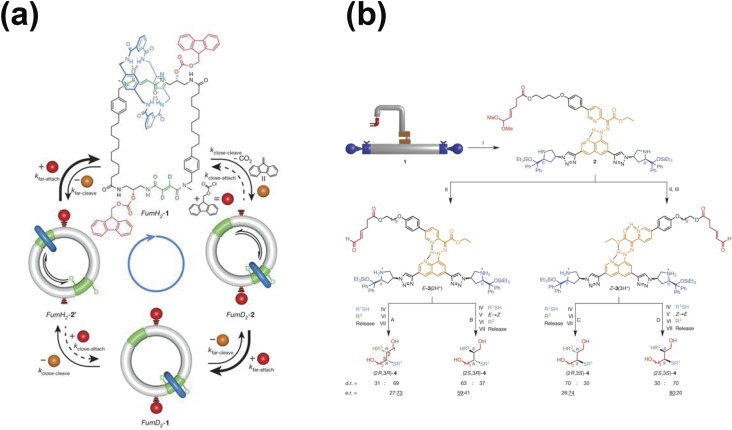
Non-biological molecular robots. (a) The operation of a continuous chemically fueled catenane rotary motor. The smaller ring (blue) travels clockwise around the larger ring (gray) when the fuel (red) is present. Reproduced with permission from Wilson *et al.*, Nature **534**(7606), 235–240 (2016). Copyright 2016 Springer Nature. (b) The operation of a molecular assembler. The molecular assembler produces four stereoisomers of a compound by different sequences of chemical addition and arm switching movements. Reproduced with permission from Kassem *et al.*, Nature **549**(7672), 374–378 (2017). Copyright 2017 Springer Nature.

Seeking to emulate how ATP, a single chemical fuel, can power molecular machines inside of cells, Wilson and co-workers introduced a catenane-based molecular motor that continuously moves as long as a chemical fuel, 9-fluorenylmethoxycarbonyl chloride, is present.^15^ Figure 4(a) presents the operation mechanism of the chemically fueled catenane rotary motor. Based on a catenane structure, a smaller ring consisting of a benzylic amide macrocycle (shown in blue) on the rotary motor traveled clockwise around the larger ring. On the larger ring, there were two fumaramide residues (shown in green) serving as binding sites for the smaller ring. The chemical fuel (shown in red) can react with the larger ring and sterically block the passage of the smaller ring, trapping the smaller ring on one fumaramide site or the other. The removal of the chemical fuel enabled the smaller rings to travel along the track via Brownian motion until the next attachment of the fuel. To create a directional bias, the researchers designed different reaction rates of fuel attachment and removal. The fuel attachment rate was dependent on the position of the ring, while the removal rate was independent of the smaller ring position. Due to this difference, the smaller ring can only travel clockwise around the larger ring despite Brownian motion occurring in both directions when the smaller ring was trapped. However, to inverse the directionality of the design requires substantial chemical modifications. To that end, Foy and co-workers have introduced modulator subunits in combination with unidirectional light-driven rotary motors, enabling the reversal of their integrated motions.^18^

While catenanes allow rotatory motions, the ring on rotaxanes can shuttle along the axle molecule. This property is used to create molecular switches to regulate catalysis events, switching on and off a catalyst by varying added chemical fuels.^19^ In addition to shuttling between two stops, Leigh and co-workers leveraged the linear movement of the ring on rotaxanes to sequentially synthesize peptides from amino acids. Amino acids were placed along with the axle molecules. The ring on the rotaxanes traveled along the axle molecule while synthesizing peptides in the order of amino acids picked up by the ring.^20^ Similarly, Cheng and co-workers utilized a rotaxane-based structure to transport small molecules as an artificial molecular pump.^16^ When supplied with redox energy, the artificial pump can transport cargo from a relatively low concentration state to a high concentration state. These catenane- and rotaxane-based machines consist of two mechanically interlocked molecules. They demonstrate how simple molecules can be integrated to work together to form molecular machines using a single energy input.

Molecular robots are not limited to catenane- and rotaxane-based structures. Kassem and co-workers created a molecular assembler that moves substrates between various activating sites to synthesize different products^21^ [Fig. 4(b)]. The molecular robot arm in the assembler can grip and release specific substrates. The robot platform in the assembler contains two reactive sites that are spatially distinct but chemically similar. The rotary switch connecting the arm and the platform steers the molecular robot arm between two modes—left-handed mode and right-handed mode—via the addition or removal of a proton. The multistep assembly process begins when a substrate is loaded onto the arm. The molecular robot arm moves the substrate around, putting it in different activated sites. Depending on the sequence of chemical addition and arm switching movements, the molecular assembler can generate four stereoisomers of a compound in a sequential one-pot reaction. The molecular assembler offers selective synthesis of diastereoisomers, which is not possible using conventional iminium–enamine organocatalysis. Furthermore, the molecular assembler allows for streamlining organic synthesis without the need for purification after each assembly step.

Biological molecular robots exploit the characteristics of biological molecules as actuators and perception-action loops. For example, Valero and co-workers employed transcription machines to build a DNA nanoengine.^22^ Similar to catenane-based motors, the nanoengine consisted of two interlocked DNA rings. An engineered DNA polymerase can attach to the DNA rings and produce RNA transcripts that are used to guide the machine movement along predefined DNA tracks.^22^ Another example is to utilize protein–protein interactions. By exploiting the interaction between ligands and cell-surface receptors, García-López and co-workers built a ligand-attached molecular machine that can drill through the target cells’ membrane at specific regions.^23^

Because of its unique sequence-dictated structural and functional features, DNA has been widely adopted to construct molecular robots. Their selective and sensitive responses to small molecules, proteins, and nucleic acids allow DNA structures to be responsive to various input molecules.^24^ Douglas and co-workers developed an autonomous molecular robot based on DNA aptamer-encoded logic gates, enabling it to respond to a wide array of inputs.^25^ When the autonomous robot perceived environmental cues, the robot processed inputs according to implemented logic gates and decided whether or not to drop off payloads.^25^

Furthermore, DNA is highly stable and programmable, allowing precise and predictable nanostructure designs via base-pairing rules. By designing a sequence of DNA building blocks, these DNA fragments can self-assemble into almost any arbitrary structure on the nanoscale level. This process is called DNA origami. Through dynamic interactions between building blocks, these DNA structures can change shapes in response to input stimuli via sequence-specific binding.^26^ For example, via DNA origami, DNA-assembled multicomponent systems imitating macroscopic gear trains, such as rack-and-pinion gearing and epicyclic gearing, can be produced at nanoscale.^27^

**FIG. 5.**
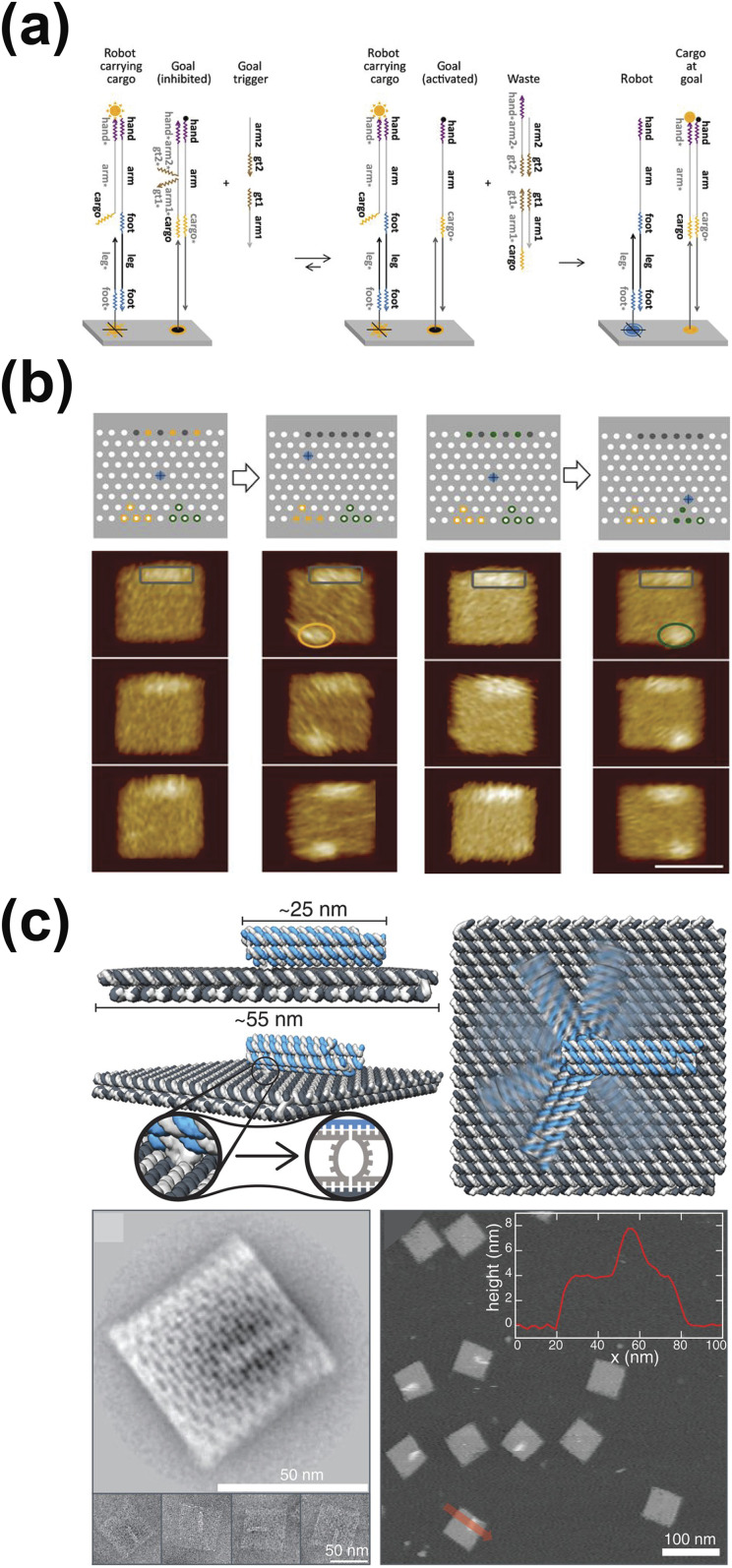
Biological molecular robots. (a) Mechanism of DNA robots sorting cargo. (b) AFM images of cargo sorting results. The robot (blue) sorted six cargoes into two categories (yellow and green). The scale bar is 50 nm. [(a) and (b)] Reproduced with permission from Reproduced with permission from Thubagere *et al.*, Science **357**(6356), eaan6558 (2017). Copyright 2017 The American Association for the Advancement of Science. (c) A molecular robot arm whose rotation can be controlled by externally applied electric fields. Schematics of the DNA molecular arm are shown as side (top left) and top views (top right). The bottom left panel shows the TEM images of the DNA molecular arm in top view. The bottom right panel shows the AFM images of the molecular robot arm in top view with the height profile measured along the direction by the red arrow. Reproduced with permission from Kopperger *et al.*, Science **359**(6373), 296–301 (2018). Copyright 2018 The American Association for the Advancement of Science.

In addition to forming arbitrary structures, the base-pairing rule can be used to implement perception-action behaviors in autonomous molecular robots. Qian and co-workers developed an autonomous DNA robot capable of performing cargo-sorting tasks.^28^ Sorting cargo is a complicated task, including steps like picking up the cargo, recognizing it, and discarding it in the correct storage place. Composed of one arm, one hand, and a single-stranded DNA walker, the DNA robot can stroll around a DNA origami surface via a reversible strand-displacement reaction [Fig. 5(a)]. While exploring the DNA origami surface, the robot picked up different encountered cargo and delivered them to designated areas via an irreversible strand-displacement reaction between the robot and surface. After dropping off the cargo, the DNA robot kept walking around randomly and repeated the process until all cargo was sorted out. In this way, the robot perceived environment cues, analyzed inputs, and made actions all based on algorithms implemented via the base-pairing property. To test the robot, they put it on DNA origami surfaces with six disorganized cargoes. The robot sorted six molecular cargoes into two categories and put the cargoes at correct locations within 24 h [Fig. 5(b)]. The researchers suggested that multiple DNA robots working simultaneously can reduce the task-completion time. The cargo-sorting DNA robots have potential applications in manufacturing molecular devices, such as molecular robots.

Even though sequence-specific actuation can be precisely designed, DNA hybridization is a slow process. To increase translational speed, recently, Bazrafshan and co-workers developed a DNA motor that can run at up to 100 nm/min, which is ten times faster than previous motors.^29^ Kopperger and co-workers built a DNA origami robot arm that can be directly controlled by external applied electric fields to bypass the slow DNA hybridization process^30^ [Fig. 5(c)]. The 25 nm-long robot arm was made out of DNA double helices bundled together. The robot arm was placed on a 55 × 55 nm^2^ DNA origami plate. The arm was connected to the plate via a flexible single-stranded DNA, allowing the arm to rotate freely relative to the platform. Since DNA is a charged molecule, DNA moved in response to applied electric fields. The researchers utilized this property to control the DNA robot arm with an externally applied electric field. Protruding single-stranded DNA monomers were placed on the platform to latch down the molecular robot arm temporarily, achieving precise control of the DNA arm. As a result, a computer-controlled electric field switched the molecular robot arm between predefined positions within milliseconds. Next, the team used the developed molecular robot arm to transport molecules and nanoparticles over tens of nanometers, which can be useful for controlling photonic and plasmonic processes. The team proposed that adopting nanostructure electrodes may enable control over individual robot arms, which have the potential to become molecular mechanical memory.

## CELL-FREE SYNTHETIC BIOLOGY AND MOLECULAR ROBOTS

Most of the above-mentioned DNA molecular robots utilize base-pairing properties to create actuation and perception-action behaviors, while encoded genetic information remains underexplored. Repurposing and reprogramming molecular modules, synthetic biology has utilized the encoded genetic information to construct perception-action behaviors on living organisms, generating designed outputs in response to specific inputs. Perception-action behaviors are programmed in synthetic genetic circuits. Transcription and translation processes drive synthetic circuits and generate output, such as proteins, according to encoded algorithms.

Inspired by electrical engineering, synthetic biology has developed a variety of genetic circuits reminiscent of electronic circuits in biological systems, such as the toggle switch.^31^ Since logic gates are the fundamental building blocks of digital electronics, essential Boolean logic gates and memory units were some of the first synthetic circuits created in living cells.^32,33^ Next, digital circuits such as counters^34^ were constructed in cells, paving the way to realize computational devices in biological systems. Recently, a protein-based central processing unit (CPU) was demonstrated to run multiple molecular algorithms including binary arithmetic, which provides the potential to do large-scale biocomputing inside cells.^35^

The use of whole-cells as the chassis requires laborious genetic engineering and suffers from unpredictable interplays between designed and natural systems due to the complex environment within living organisms.^36^ Cell-free synthetic biology provides a platform to execute these circuits without the limitations mentioned above. Consisting of molecular machinery extracted from cells, cell-free systems were initially designed for *in vitro* protein synthesis. Cell-free systems contain essential enzymes for transcription and translation, allowing the synthetic genetic circuit to be transcribed and translated without cells. Furthermore, the flexibility of cell-free platforms enables the customization and optimization of reactions, such as adding proteins or small molecules to improve the synthetic genetic circuit performance.^37^ A holistic approach was recently developed to optimize cell-free platforms and yielded a higher protein expression level than cell-free systems produced prior to optimization.^38^ Hence, cell-free platforms offer an ideal test-bed for developing genetic circuits and, potentially, for controlling molecular robots.

Logic-operating synthetic genetic circuits are also available in cell-free platforms. Even though some synthetic genetic circuits may be limited to specific cell types, recent developments of synthetic genetic circuits improve transferability. Chen and co-workers developed a set of logic gates including gates with multiple inputs that function in cell-free systems, yeast, and human cells.^39^ These protein-based logic gates enable faster responses than synthetic circuits based on transcription systems. Additionally, the tunable nature of cell-free platforms enables modification to mirror a specific cell environment, rendering the possibility to execute genetic circuits that are not designed for cell-free platforms.^37^ Moreover, genetic circuits can be directly added to cell-free reactions at desired concentrations, providing precise control over the gene expression by eliminating endogenous variables introduced while putting genetic constructs into cells.^40^

**FIG. 6.**
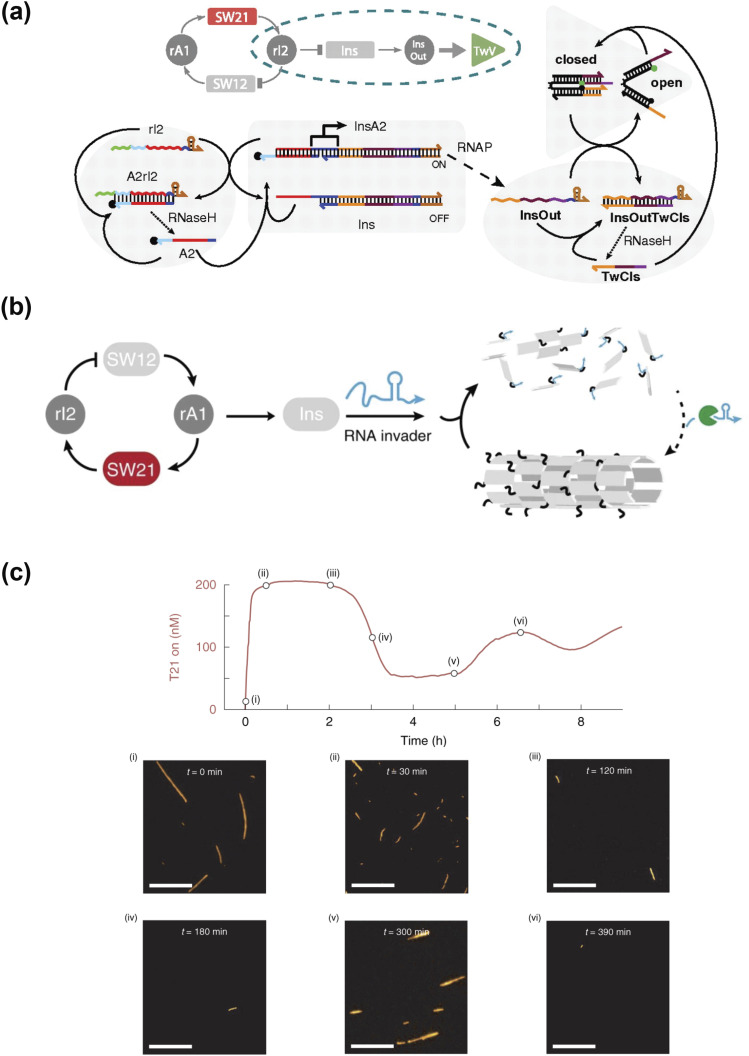
Using cell-free synthetic tools to drive molecular robot behaviors. A synthetic genetic oscillator controlled (a) DNA tweezers and (b) DNA nanostructure assembly. (c) Images of the nanotube assembly at various time points. Few short nanotubes are visible during the oscillator peaks [point (ii)–(iv)], while long nanotubes can be found at the oscillator wells [point (v)]. The scale bar is 10 *μ*m. Reproduced with permission from Franco *et al.*, Proc. Natl. Acad Sci. U. S. A. **108**(40), E784–E793 (2011). Copyright 2011 National Academy of Science and Green *et al.*, Nat. Chem. **11**(6), 510–520 (2019). Copyright 2019 Springer Nature.

Cell-free platforms provide a new paradigm for building autonomous molecular robots with actuator and perception-action behaviors. For example, Franco and co-workers utilized a cell-free oscillator to operate a DNA-based nanomechanical device termed the DNA tweezer^41^ [Fig. 6(a)]. Comprised of two double-helical domains connected by a hinge, this DNA tweezer has two single-stranded areas capable of binding to their individual target and, therefore, closes the tweezer. While oscillators generate clock signals in electronics, oscillators in cells control the timing of cellular processes. To create a molecular clock for timing downstream events, the team turned to synthetic circuits and cell-free platforms. The synthetic circuits in this work consisted of gene templates called genelets, which were used to transcribe RNA molecules. A simple oscillator circuit consisted of genelets SW21 and SW12 with an inhibiting RNA, rI2, and an activating RNA, rA1. When switch SW 21 was ON, the cell-free platform transcribed an inhibiting RNA, rI2. The rI2 inhibited transcription of switch SW12 by removing part of its promoter region. rI2 turned switch SW12 off, resulting in no transcription of activating RNA, rA1. The activating RNA, rA1, activated transcription from SW21 by releasing the promotor region in SW21.

To minimize the downstream load effect on the oscillator circuit, the researchers added an insulator to the genetic circuit reminiscent of an amplifier stage in electric circuits. The insulator genelets, Ins, operated in parallel with an oscillator switch SW12, which allowed for Ins activation by A2 and inhibition by rI2. The insulator produced a new RNA species, InsOut, which opened the tweezers previously closed by the DNA strand, TwCls. Next, the TwCls·InsOut complex was degraded, creating free TwCIs. The insulator stage enabled the oscillator to drive the opening and closing of more tweezers while isolating tweezer operation from oscillators. Recently, Green and co-workers used a similar cell-free oscillator module to control the DNA molecular machine self-assembly^42^ [Fig. 6(b)]. As building blocks, DNA double-crossover tiles could self-assemble into a DNA nanotube. On each building block, there was a single strand area that was able to bind to an invader strand. The tile-invader complex resulted in the disassembly of DNA nanotubes. In this oscillator, the insulator produced the invader strand that caused the disassembly of DNA nanorobots. In Fig. 6(c), the images show the assembly and disassembly process with the oscillation of the RNA invader. These projects demonstrate using cell-free synthetic tools to drive molecular robot behaviors with high-level complexity.

Instead of using cell-free circuits to drive perception-action behaviors, Hamada and co-workers developed an onboard metabolism system on DNA robots with a cell-free platform.^43^ Through the cell-free based artificial metabolism, a DNA material can autonomously self-assemble and disassemble, like a living organism growing and decaying. Even though cell-free platforms were not directly used in this work, Gines and co-workers proposed to build memory, perception-action, and communication via putting synthetic DNA circuits on microparticles in an enzymatic solution similar to the cell-free platform.^44^ In this way, Gines and co-workers were able to produce collective behaviors among microparticles, such as retrieving information over long distances.

Despite all the exciting developments, synthetic biology is confined to lab settings due to the need to maintain reactions at specific conditions. Cell-free systems offer a way out. Cell-free platforms can be freeze-dried and stored at room temperature. At the time of need, simply adding water to the freeze-dried cell-free solution can activate it. This feature offers solutions to deploying synthetic genetic tools in the field for applications, including diagnostics and biomanufacturing.^36^

**FIG. 7.**
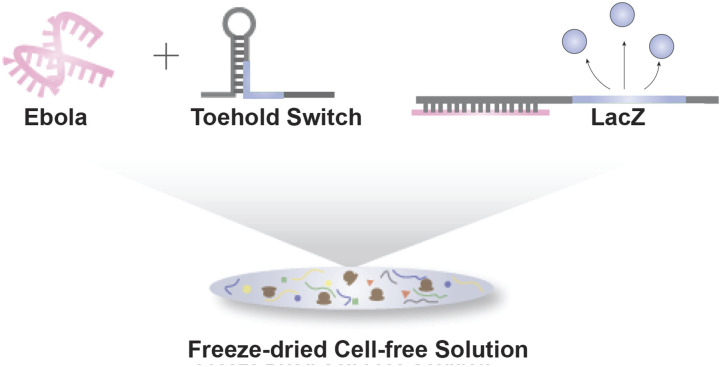
A freeze-dried paper-based cell-free platform for *in vitro* Ebola diagnostics. (a) A schematic of Ebola biosensors in a freeze-dried, paper-based cell-free platform. Synthetic biology-based biosensors, toehold switches, are employed to detect Ebola. A freeze-dried paper-based cell-free platform carries out the detection and generates LacZ as its output signal.

In order to develop molecular robots that can be more broadly deployed outside of the laboratory, an ability to manufacture these robots at the point-of-use would be ideal. Fortunately, portability is one of the key benefits of using a cell-free synthetic biology approach. For example, Pardee and co-workers successfully moved synthetic biology outside of the lab by embedding the freeze-dried cell-free solution and genetic constructs onto paper.^45^ This construct was stable at room temperature and readily stored and distributed to the field. The team deployed the cell-free platform and DNA elements within 2 mm diameter filter paper discs and lyophilized overnight. After lyophilizing, these paper discs were rehydrated with water, and the cell-free reactions were successfully carried out on the paper discs. Using this paper-based, cell-free platform, the researchers built strain-specific Ebola sensors for *in vitro* diagnostics^45^ [Fig. 7]. In addition to using the paper-based cell-free platform for diagnostics, the team applied the paper-based, cell-free platform to on-site, on-demand biomolecular manufacturing, such as for antimicrobial peptide and vaccine production.^46^ Apart from diagnostic and therapeutic applications, the lyophilized cell-free platform can also be used as educational tools to explore synthetic biology circuits in the classroom.^47^ Ultimately, the team demonstrated that a stable and abiotic cell-free platform for synthetic biology that can also be used with materials other than paper. The lyophilized cell-free platform could one day be used to implement perception-action behaviors with molecular robots outside of lab environments.

## SYNTHETIC BIOLOGY AND ROBOTICS

**FIG. 8.**
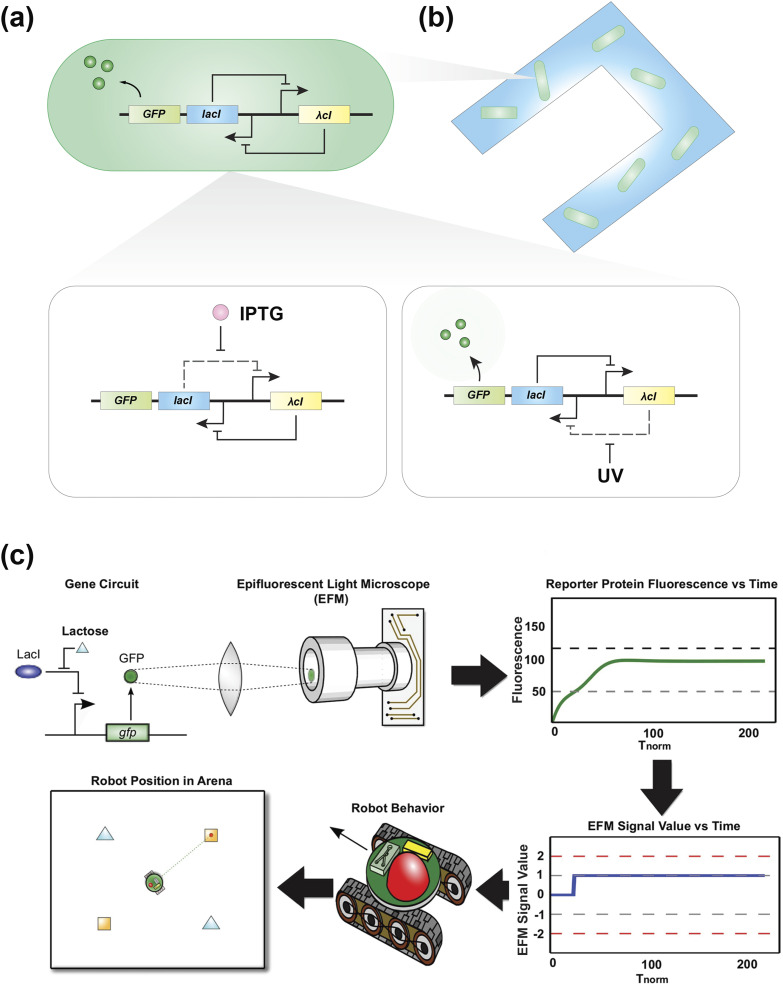
Genetically engineered bacteria as perception-action modules for robots at different scales. (a) A schematic of a genetic toggle switch circuit activated by different chemical inducers and the corresponding activity of its regulatory genes, *λcI* and *lacI*. (b) A microrobot with genetically engineered bacteria that sense UV light and respond by generating GFP. (c) A simulated model of engineered bacteria that function as perception-action modules to drive a macroscale robot. Genetic circuits generate fluorescence in response to environmental cues. A simulated onboard microscope measures the fluorescence signal and activates motion algorithms for a macroscale robot in response. The units for fluorescence and EFM signal values are both absolute units. Reproduced with permission from K. C. Heyde and W. C. Ruder, Sci. Rep. **5**(1), 11988 (2015). Copyright 2015 Springer Nature.

Synthetic biology has been adopted to provide perception-action behaviors in robots at macroscale and microscale. Apart from the cardiomyocytes in the robotic ray discussed earlier,^13^ genetically engineered skeletal muscle^48,49^ and bacteria^50,51^ have been used as perception-action modules for robots. Furthermore, Steager and co-workers utilized genetic toggle switches as sensors, signal processors, and memory units in microrobots.^52^ Figure 8(a) illustrates a schematic of a genetic toggle switch circuit under different inducer combinations and the corresponding activity of gene products. In this genetic toggle switch, the green fluorescent protein (GFP) was synthetically engineered in parallel with *lacI* transcription and used as an optical reporter molecule. Isopropyl-β-D-thiogalactoside (IPTG) repressed protein LacR (transcripted and translated from *lacI*), which induces the production of protein λcI. Protein λcI repressed GFP and LacR production. When UV light was introduced to the toggle switch, it caused DNA damage and led to enhanced λcI protein degradation, and therefore, rescued GFP production. The researchers incorporated engineered bacteria carrying this genetic toggle switch with microrobots and deployed them to detect UV light in a workspace [Fig. 8(b)]. When microrobots returned to base, the engineered bacteria onboard reported whether the visited area was exposed to UV light by the green fluorescence readout.^52^

Apart from using genetic toggle switches in microrobots, macroscale robots can adopt synthetic biology tools as their perception-action loop modules. Heyde and Ruder proposed using engineered bacteria equipped with genetic toggle switches to maneuver a biomimetic, macroscale robot^12^ [Fig. 8(c)]. Inspired by microbiome–host interactions, they created an *in silico* model of a microbiome consisting of engineered cells carrying synthetic genetic circuits, together with a robotic host housing an onboard microfluidic chemostat and microscope. The onboard microfluidic chemostat was used to mimic a microbiome environment within an organism. Heyde and Ruder used this system to simulate how various genetically engineered bacteria could affect the robot behavior. With the increased complexity of genetic circuits, a variety of behaviors emerged, such as stalk-pause-strike predation. Their model provides a tool to investigate host–microbiome interactions and offers a novel paradigm to create perception-action loops in autonomous robots. These examples of robots at the macroscale and microscale with synthetic biology-based perception-action modules reveal how synthetic biological circuits could be deployed with robotic counterparts at the molecular level.

## OUTLOOK

From macroscale to molecular robotics, robots at various scales have taken advantage of synthetic biology tools to achieve complex perception-action behaviors. Synthetic biology-based perception-action modules are of particular interest to molecular robots since they can be readily integrated at the molecular scale. Embarking from catenanes and rotaxanes, molecular machines have gradually evolved into molecular robots capable of more complex tasks.

Compared to their macroscale counterparts, there is a considerable gap between molecular machines and molecular robots. Similar to the synergy between bioelectronics and soft robotics, synthetic biology offers perception-action modules, which we believe can fill the gap between molecular machines and molecular robots. We have highlighted a myriad of synthetic genetic circuits reminiscent of electronic digital circuits available to engineer molecular robots. The complexity of molecular robot behaviors can improve with advances in biocomputing synthetic genetic circuits. At present, synthetic biologists have gene-based versions of CPU. In the future, synthetic circuits can confer intelligence and become the control system for autonomous molecular robots.

Cell-free systems power synthetic genetic circuits without reliance on living cells, extending synthetic biology into the real world. If molecular robots adopt synthetic biology-based tools as their perception-action modules, their movements and functions will no longer be confined to specific reaction criteria. Cell-free systems have freed synthetic biology from lab settings. Likewise, cell-free platforms can help molecular robots step outside the laboratory in the future.

Biological components have changed the actuation system of robots at small scales.^53^ Exploiting unique characteristics of bacteria and cells, microscale biohybrid robots can autonomously actuate, deliver cargo, and serve as novel therapies.^54,55^ While bacteria and cells are too large for molecular robots, biological molecules such as DNA and proteins are at the scale for the integration of molecular robots. With the current progress in DNA origami, DNA is an ideal candidate as an actuator in molecular robots because of its high stability, programmability, and modularity.

Furthermore, DNA based actuators and sensors can be integrated with molecular robots consisting of carbon nanotubes and magnetic nanoparticles. Systems integrating biomolecules and carbon nanotubes have been used as biosensors to build complex nanostructures for nanobioelectronics.^56^ The unique electrical and mechanical properties of carbon nanotubes have made them exciting candidates to be used as the molecular robot chassis. At the same time, magnetic particles as the molecular robot chassis enable the contactless control of robots via externally applied magnetic fields.^57,58^ Magnetic control is a promising method for steering molecular robots for medical applications due to its efficiency, contactless nature, precision, and the established safety of penetrating the human body with magnetic fields (e.g., MRI). Leveraging the capability of synthetic biology and cell-free platforms in creating complex perception-action behaviors, molecular robots can acquire the ability to sense, analyze, and respond to complex environments.

**FIG. 9.**
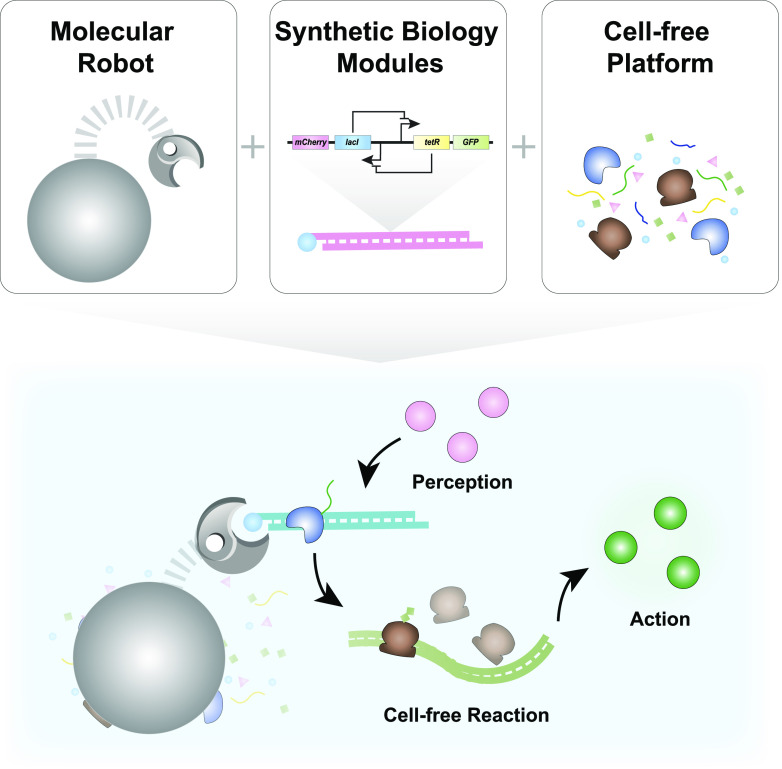
Next generation molecular robots. An envisioned biohybrid molecular robot with synthetic biology modules that operates in cell-free environments.

Ultimately, we envision that the next generation of molecular robots with advanced autonomy will be biohybrid robots containing synthetic biology-based perception-action modules encoded within DNA molecules. For example, a magnetic particle based molecular robot could be anchored to DNA molecules encoding synthetic biology-based perception-action modules [Fig. 9]. The magnetic property would allow for remote control and actuation of robots via externally applied magnetic fields. The synthetic biology-based perception-action modules would provide readily available sensor modules and sophisticated molecular algorithms. Cell-free platforms would provide transcription and translation machinery to power on-board synthetic biology perception-action modules and carry out designed behaviors. Ultimately, these advanced behaviors would result in molecular robots with autonomy. These robots would be a promising technology in biomedical applications, such as active drug delivery and point-of-care diagnostics.

## DATA AVAILABILITY

Data sharing is not applicable to this article as no new data were created or analyzed in this study.
